# Plant growth-promoting traits and biocontrol mechanism of three *Pseudomonas* strains against *Geotrichum citri-aurantii*

**DOI:** 10.3389/fmicb.2026.1970166

**Published:** 2026-10-26

**Authors:** Chunling Wang, Lutian Mao, Boshi Zhu, Xing Liu, Xinjian Xv, Jiaxin Zhuo

**Affiliations:** College of Life Science, Huizhou University, Huizhou, China

**Keywords:** plant growth-promoting traits, antagonistic activity, *Geotrichum citri-aurantii*, genomic analysis, T6SS

## Abstract

**Background:**

Plant growth-promoting rhizobacteria (PGPR) play essential roles in improving plant growth performance and enhancing plant disease resistance. *Pseudomonas* species are widely recognized as promising beneficial microorganisms owing to their excellent plant growth-promoting and biocontrol potential.

**Methods:**

Three bacterial strains, MBJ003, MBJ016 and MB1046, were isolated from potato and tomato rhizosphere soils collected in Guangdong Province, P.R. China. Taxonomic identification was performed based on morphological characteristics, physiological and biochemical profiles, as well as phylogenetic and whole-genome analyses. Genomic features, plant-growth promoting traits and antifungal activities against *Geotrichum citri-aurantii* were characterized, and the function of the type VI secretion system (T6SS) in strain MB1046 was explored.

**Results:**

Strain MBJ003 was identified as *Pseudomonas paraeruginosa*, MB1046 as *Pseudomonas palmensis*, and MBJ016 represents a novel *Pseudomonas* species. Whole-genome sequencing uncovered abundant and diverse secondary metabolite biosynthetic gene clusters (BGCs) underlying biocontrol capacity. Additionally, strain MB1046 harbours 17 type VI secretion system (T6SS) components. All three strains exhibited multiple plant-beneficial traits including phosphate solubilization, siderophore synthesis and IAA production, while strain MBJ003 additionally showed protease activity. The three strains displayed strong antagonism toward *G. citri-aurantii*. Their cell-free fermentation supernatants exerted antifungal effects, with MBJ003 showing the strongest inhibition. In vitro inoculation assays confirmed that these strains effectively control citrus postharvest disease caused by *G. citri-aurantii*. We further provided insights into the T6SS-mediated antagonism of MB1046 against *G. citri-aurantii*.

**Conclusion:**

This study systematically characterizes the plant-growth-promoting properties and antifungal mechanism of three *Pseudomonas* strains. These strains are valuable microbial candidates for developing biocontrol agents to manage crop postharvest diseases.

## 1 Introduction

Plant growth-promoting rhizobacteria (PGPR) are beneficial root-colonizing microorganisms that exert dual agroecological functions in facilitating plant growth and suppressing phytopathogens (Kumari et al., 2024; Stanzin et al., 2017). Among diverse PGPR populations, *Pseudomonas* species, including *Pseudomonas paraeruginosa, Pseudomonas plecoglossicida*, and *Pseudomonas palmensis*, are widely recognized as microbial inoculants due to their strong environmental adaptability, stable rhizosphere colonization capability, and abundant secondary metabolic diversity (Das et al., 2023; Saroja et al., 2026; Singhmar et al., 2024). These beneficial strains improve crop growth via multiple direct regulatory mechanisms, including nutrient activation and solubilization, phytohormone synthesis, and stress tolerance enhancement (Mankoti et al., 2024). Meanwhile, they also exert indirect biocontrol effects, effectively suppressing the occurrence and prevalence of soil-borne and postharvest plant diseases (Dobrzyński and Jakubowska, 2025; Haas and Défago, 2005; Raaijmakers et al., 2010). Biocontrol bacteria can suppress phytopathogenic fungi through multiple antagonistic metabolic pathways and functional mechanisms. Phenazine-1-carboxylate and 2,4-diacetylphloroglucinol (2,4-DAPG) have been identified as effective fungicidal compounds (Haas and Défago, 2005; Zhu et al., 2018). Notably, recent evidence suggests that contact-dependent antagonistic mechanisms are widespread among the bacterial competitors, with type VI secretion system (T6SS) and type IV secretion system (T4SS) being the most commonly utilized in gram-negative bacteria (Timothy et al., 2020).

Most existing studies regarding the plant growth-promoting performance of *Pseudomonas* only focus on single functional traits or single crop application scenarios (Jesús et al., 2020; Kumar P. et al., 2016). The systematic evaluation of their growth-promoting effects, biocontrol efficacy against postharvest citrus diseases, and the molecular mechanisms against phytopathogenic fungi remain insufficiently and unsystematically elucidated. In this study, we isolated three PGPR strains from potato and tomato rhizosphere soil collected from Guangdong Province, P.R. China. A comprehensive investigation was conducted, including strain morphological and molecular identification, *in vitro* evaluation of plant growth-promoting and antifungal activities, biocontrol efficacy determination against citrus sour rot caused by *Geotrichum citri-aurantii*, whole-genome sequencing, bioinformatic analysis, construction of T6SS deletion mutant and study on its antifungal function. The findings will provide a solid theoretical basis and high-quality strain resources for the exploitation of novel microbial biofertilizers and biocontrol agents in green agriculture.

## 2 Materials and methods

### 2.1 Strains isolation and preservation

Three bacterial strains with inorganic phosphorus-solubilizing capacity were obtained in the study. Strain MB1046 was isolated from potato rhizosphere soil collected in Huizhou (114 °5′05″ E; 22 °43′23″ N), and, strains MBJ003 and MBJ016 were isolated from tomato rhizosphere soil collected in Guangzhou (113 °26′26″ E; 23 °22′49″ N), Guangdong Province, P.R. China. The standard dilution plate method on Pikovskaya's agar medium (PKO, Haibo, Qingdao, China) was used for bacterial isolation. Plates were cultivated at 30 °C for 3–5 d. Single-colonies with transparent halos were picked out and purified repeatedly. Upon purification, strains were preserved at −80 °C as a suspension containing 25% v/v glycerol.

### 2.2 Identification of three strains

**Physicochemical characteristics:** colony and cell morphological characteristics cultivated on Nutrient agar (NA, Haibo, Qingdao, China) for 2–3 d at 30 °C were observed, and then cell ultrastructures were visualized using a scanning electron microscope (SEM). Gram-staining was performed using the bioMérieux Gram stain kit according to the manufacturer's instructions. Oxidase activity was performed using oxidase test strips [1% (w/v) tetramethylp-phenylenediamine, HKM] as described by Lin et al. (2014). Catalase activity was examined by bubble production after mixing cells with 3% H_2_O_2_. Temperature ranges (0, 5, 10, 15, 35, 37, 40, 41, 42, and 43 °C) for growth were observed by cultivating on NA agar for 2–3 d. Salt tolerance was tested in Nutrient broth (NB) media supplemented 0–7.0% (w/v) NaCl at 0.5 intervals for 2–3 d at 30 °C. pH range (4.0–12.0, at 0.5 intervals) for growth were tested in NB medium at 30 °C for 2–3 d. Indole, methyl red, Voges-Proskauer reaction (VP), and citric acid (IMVC system) were tested using the dry biochemical identification kit (Beijing Luqiao Co., Ltd., Beijing, China).

**Determination of bacterial growth curve**: three strains were individually inoculated into test tubes containing 5 mL of NB liquid medium, and cultured at 30 °C with shaking at 150 rpm. Seven time points (0, 4, 8, 12, 24, 36, and 48 h) were set for each strain with three replicates at each time point. A 1 mL aliquot was taken from each treatment for OD_600_ determination. Growth curves were plotted based on the measured OD_600_ values using OriginPro software.

**Phylogenetic tree:** genomic DNAs were extracted from fresh bacterial cells using the HiPure Bacterial DNA kit (Magen Biotech Co., Ltd., Guangzhou, China) according to the manufacturer's instructions. The 16S rRNA gene was amplified using genomic DNAs as templates with the universal primers 27F and 1492R (Kai et al., 2006). PCR products were sequenced in Majorbio, China. The obtained 16S rRNA gene sequences of MBJ003, MBJ016, and MB1046 were submitted to the National Center for Biotechnology Information (NCBI) database (https://www.ncbi.nlm.nih.gov/genome) under the accession numbers DB008031, DB008032, and DB008034, respectively. The 16S rRNA gene were aligned to the EzBiocloud database (https://eztaxon-e.ezbiocloud.net/). Multiple sequence alignments were analyzed using MEGA 7.0 software (Kumar S. et al., 2016). Phylogenetic tree was constructed based on 16S rRNA gene sequences using the maximum-likelihood (ML) method (Guindon and Gascuel, 2003). The topology in the phylogenetic tree was calculated based on 1000 replications and evolution distances were calculated using Kimura's two-parameter model (Kimura, 1980).

**Antibiotic resistance screening assay:** NA plates supplemented individually with 50 mg L^−1^ ampicillin, kanamycin, or tetracycline hydrochloride were prepared. The three tested strains were streaked onto these antibiotic-containing plates and incubated at 30 °C for 5 d. Bacterial growth was visually evaluated after incubation. Colonies formed on antibiotic-containing plates were considered resistant and no visible growth were regarded as susceptible. All assays were performed in triplicate.

### 2.3 Genome sequencing, assembly, and annotation of three strains

Genomic DNAs were extracted using the method described in Section 2.2, Phylogenetic tree. Illumina paired-end genomic libraries were constructed using the qualified DNA (at least 1 μg) with NEXTflex™ Rapid DNA-seq Kit (Bio, Boston, MA, USA). Whole-genome sequencing was performed on the Illumina HiSeq platform with a paired-end 150 bp (PE150) sequencing strategy at Shanghai Majorbio Bio-Pharm Technology Co., Ltd. (Shanghai, China). Low-quality reads and adapters were removed, and high-quality reads were assembled into contigs and scaffolds using SPAdes v3.11.1 with default parameters (Bankevich et al., 2012). The integrity and contamination of the genomes were evaluated using CheckM v1.1.2 (Parks et al., 2015).

Genomic functional annotation was performed via the Rapid Annotation using Subsystem Technology (RAST) pipeline (Aziz et al., 2008) with default settings. Secondary metabolite biosynthetic gene clusters (BGCs) were predicted and annotated using antiSMASH bacterial version 6.1.1 (Blin et al., 2019) under default parameters. The T6SS was reconstructed by uploading protein files to the Kyoto Encyclopedia of Genes and Genomes (KEGG) server using BlastKOALA to retrieve KEGG ortholog (KO) numbers (Kanehisa et al., 2015). The average nucleotide identity (ANI) values were estimated using OrthANIu; the digital DNA–DNA hybridization (dDDH) values were calculated using the Genome-to-Genome Distance Calculator (version 3.0; Chun et al., 2018).

### 2.4 Assessment of plant-growth-promoting activities of three strains

The inorganic phosphorus-solubilizing ability of the three strains was re-verified on PKO medium at 30 °C for 3 d (Wang et al., 2022), and the soluble phosphorus content was quantified using the molybdenum-antimony anti-colorimetric method in Pikovskaya's liquid medium, with tricalcium phosphate (Ca_3_(PO_4_)_2_) as the sole insoluble inorganic phosphorus source as previously described (Murphy and Riley, 1962). The potassium-solubilizing capacity was assessed on a medium containing potash feldspar as an insoluble potassium source at 30 °C for 3 d. Transparent halos around colonies indicated positive potassium solubilization (Hu et al., 2006). Siderophore production was qualitatively detected on chrome azurol S (CAS) plates at 30 °C for 3 d. Change in the color around the colonies as siderophore secretion (Schwyn and Neilands, 1987). Indole acetic acid (IAA) production was examined using NB medium containing L-tryptophan (100 mg/mL). Briefly, 100 μL cell-free supernatant was added in a 96-well plate, and 100 μL Salkowski's reagent was added. After incubating the reaction mixture in the dark for 30 min, the absorbance OD_530nm_ was measured using a microplate reader. A standard curve was made using 0–20 mg/L IAA, and the contents of IAA produced were calculated (Glickmann and Dessaux, 1995). Protease activity was determined via the Folin phenol method per GB/T 23527-2009 standard after incubation at 30 °C for 3 d.

### 2.5 Biocontrol assays of three strains against *G. citri-aurantii*

#### 2.5.1 Antagonistic activity

Plate confrontation assays were employed to evaluate the antagonistic activities of the three strains against *G. citri-aurantii* TSF19. Briefly, a 5-mm-diameter block of TSF19 was inoculated at the center of each NA plate. Subsequently, each of the three strains were spot-inoculated on individual plates, with four spots 1.0 cm from the fungal plug per plate. The control treatment was NA plate inoculated with only TSF19. After incubation at 30 °C for 7 d, the mycelial growth diameter of TSF19 was measured to calculate the antifungal inhibition rate. Three biological replications were performed.

The antifungal activity of bacterial fermentation supernatants was further determined. The three strains were cultured in NB medium at 30 °C for 2 d. Fermentation supernatants were collected by centrifugation and filtered using 0.22 μm Polyvinylidene fluoride (PVDF, Delvstiab, China) filters. The filtered supernatants were mixed with NA medium at a final volume concentration of 20%. A 5-mm-diameter TSF19 mycelial plug was placed at the center of each plate, followed by incubation at 30 °C for 5 d. TSF19 mycelial plug was inoculated on NA medium supplemented with 20% NB medium to serve as the control. The fungal colony diameter was measured to evaluate the antifungal inhibition rate. Three biological replicates were set for each treatment.

The antifungal inhibition rate was calculated using the following formula:

Inhibition rate (%) = [(Colony diameter of control (mm) – Colony diameter of treatment (mm)) / Colony diameter of control (mm)] × 100%

#### 2.5.2 Biocontrol efficacy against citrus sour rot

Citrus fruits with uniform size and no mechanical damage were selected to evaluate the biocontrol efficacy against citrus sour rot caused by *G. citri-aurantii*. The three bacterial strains were cultured in 500 mL NB media at 30 °C with shaking at 150 rpm for 2 d, respectively. The bacterial suspensions were adjusted to OD_600_ of 1.0. Citrus fruit surfaces were rinsed with purified water and disinfected sequentially with 2.5% NaClO for 30 s, 75% ethanol for 30 s, and sterile water for 60 s. After complete air-drying, a cross-shaped wound was created on the surface of each fruit. The treated fruits were immersed in different bacterial fermentation broths (OD_600_ = 1.0) for 90 s and air-dried again. Fruits immersed in NB medium served as the control. Subsequently, 2 μL of *G. citri-aurantii* spore suspension (200 conidia/μL) was injected into each wound. Five citrus fruits were used for each treatment. After inoculation, fruits were cultured at 30 °C and 85% relative humidity for 3 days.

Control efficacy was calculated according to the following formula:

Lesion area = π × [(lesion length + lesion width) / 2]^2^

Control efficacy (%) = [(Lesion area of control group – Lesion area of treatment group) / Lesion area of control group] × 100%

### 2.6 In-frame deletion mutant construction

To investigate the role of T6SS in the antifungal activity of *P. palmensis* MB1046, an in-frame deletion mutant of the T6SS effector gene *vgrG* (IPAKHDLA_02735) was constructed. All strains, plasmids, and primers used in this study are listed in Supplementary Table S1. The *vgrG* deletion mutant (1,411 bp) was generated via double-crossover homologous recombination using the suicide plasmid pEX18Gm, as previously described (Feng et al., 2020; Lin et al., 2025). Briefly, the upstream and downstream flanking sequences of the *vgrG* were amplified by PCR and inserted into the pEX18Gm vector by One Step Clone Kit (Vazyme Biotech, Nanjing, China). The recombinant plasmid was transformed into DH5α competent cells via heat shock at 42 °C and subsequently introduced into strain MB1046 through triparental mating, with the helper plasmid pRK2013 providing conjugation-related functions. Single-crossover recombinants were screened on *Pseudomonas* Agar Base (CN agar, Biosense Biotech, China) plates containing 50 μg/mL gentamicin. Positive recombinants were further cultured on LB plates supplemented with 20% (w/v) sucrose to screen for double-crossover deletion mutants. The successful construction of the Δ*vgrG* mutant was verified by PCR amplification and DNA sequenced.

### 2.7 Antifungal assay

The wild-type strain MB1046 and Δ*vgrG* mutant were tested and compared for their antifungal activity. Bacterial strains were cultured overnight in NB media and harvested by centrifugation at 8,000 rpm for 5 min and washed three times with sterile double-distilled water. The cell suspension was adjusted to an OD_600_ of 1.0. The conidia of *G. citri-aurantii* were collected and filtered through two layers of Mirocloth to remove residual mycelia, then diluted to a final concentration of 200 conidia/μL with sterile double-distilled water. For the coculture assay, bacterial suspension and fungal conidia suspension were mixed at a 1:1 volume ratio, and 2 μL of the mixture was spotted on NA plates. The plates were incubated at 30 °C for 48 h, and fungal growth was observed. In the parallel spotting assay, 2 μL of bacterial suspension (OD_600_ = 1.0) and 2 μL of fungal conidia suspension were separately spotted on NA plates with a spacing of 1.0 cm between the two spots. To verify the contact-dependent antifungal mechanism, a 0.22 μm PVDF filter membrane was placed on the NA plate to physically separate bacteria and fungi. Bacterial and fungal suspensions were spotted on opposite sides of the filter membrane, with each spot 0.5 cm away from the membrane edge. The membrane permits diffusion of soluble metabolites while blocking direct physical contact between bacterial and fungal cells. All experiments were performed in three independent biological replicates.

## 3 Results

### 3.1 Identification of three *Pseudomonas* strains

The colonial morphologies of strains MBJ003, MBJ016, and MB1046 cultured on NA media are displayed in Figures 1A–C. All three strains formed circular colonies with regular margins, slight convexity, smooth and moist surfaces, and were difficult to pick. The cell morphologies of the three strains are shown in Figures 1D–F, and all cells presented a typical short rod shape. The average cell size of MBJ003 was 1.51 μm × 0.42 μm, while MBJ016 and MB1046 had cell dimensions of 1.48 μm × 0.45 μm and 1.17 μm × 0.52 μm, respectively. The growth curves of the three strains cultured in NB medium are shown in Supplementary Figure S1, and all strains exhibited consistent growth patterns with similar growth dynamics throughout the cultivation period. Phenotypic biochemical tests showed that all three strains were Gram-negative, oxidase-positive, and catalase-positive. Additional physiological and biochemical characteristics are summarized in Table 1. Antibiotic resistance assays revealed that strain MBJ003 was tolerant to 50 mg/L ampicillin, kanamycin, and tetracycline hydrochloride. In contrast, strains MBJ016 and MB1046 only exhibited resistance to ampicillin at the same concentration (Table 1).

**Figure 1:**
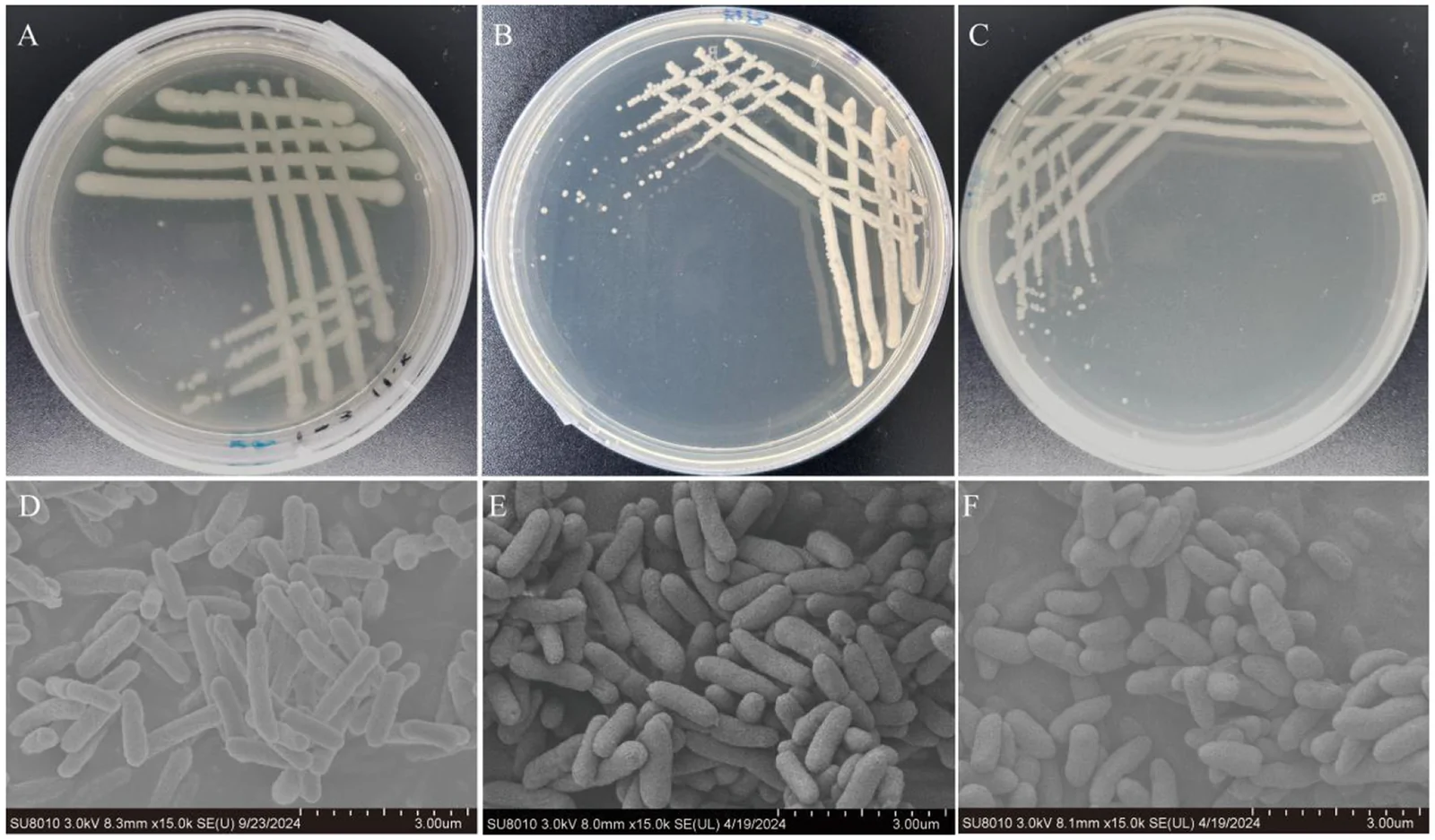
Morphological characteristics of three *Pseudomonas* strains. **(A–C)** Colonial morphology of MBJ003 **(A)**, MBJ016 **(B)**, and MB1046 **(C)** after cultivation on NA medium at 30 °C for 2 d. **(D–F)** Cell morphology of MBJ003 **(D)**, MBJ016 **(E)**, and MB1046 **(F)** observed via SEM after growth on NA medium at 30 °C for 2 d.

**Table 1:** The physiological characteristics and antibiotic tolerance of three *Pseudomonas* strains.

Characteristics	MBJ003	MBJ016	MB1046
Gram-staining reaction	–	–	–
Oxidase	+	+	+
Catalase	+	+	+
Temperature (°C)	15–42	10–41	5–35
pH value	4.5–11.5	4.5–10.5	4.5–11.0
NaCl (%, w/v)	0–6.0	0–5.5	0–6.0
Methyl red	–	–	+
Indole	–	–	–
Citric acid	+	+	+
VP	–	–	–
**Antibiotic tolerance (50 mg L** ^−1^ **)**			
Ampicillin	+	+	+
Kanamycin	+	–	–
Tetracycline	+	–	–

+ indicates positive; – indicates negative.

Phylogenetic analysis revealed that strains MBJ003, MBJ016, and MB1046 shared 100% 16S rRNA gene sequence identity with *P. paraeruginosa* PA7^T^, *P. plecoglossicida* NBRC 103162^T^, and *P. palmensis* BBB001^T^, respectively. The ML phylogenetic tree based on the 16S rRNA genes further demonstrated that these three strains clustered within the same clade as their corresponding type strains (Figure 2). The ANI and dDDH values of strain MBJ003 and *P. paraeruginosa* PA7^T^ were 99.1% and 91.7%. The ANI and dDDH values of strain MB1046 and *P. palmensis* BBB001^T^ were 99.1% and 93.8%. However, the ANI and dDDH values of strain MBJ016 and the 35 most closely related species were 79.0–93.7% and 23.2–55.1% (Supplementary Table S2), which were lower than the species boundary thresholds of 95–96% and 70%. Collectively, these results identified MBJ003 as *P. paraeruginosa*, MB1046 as *P. palmensis* and MBJ016 as a novel species of the genus *Pseudomonas*.

**Figure 2:**
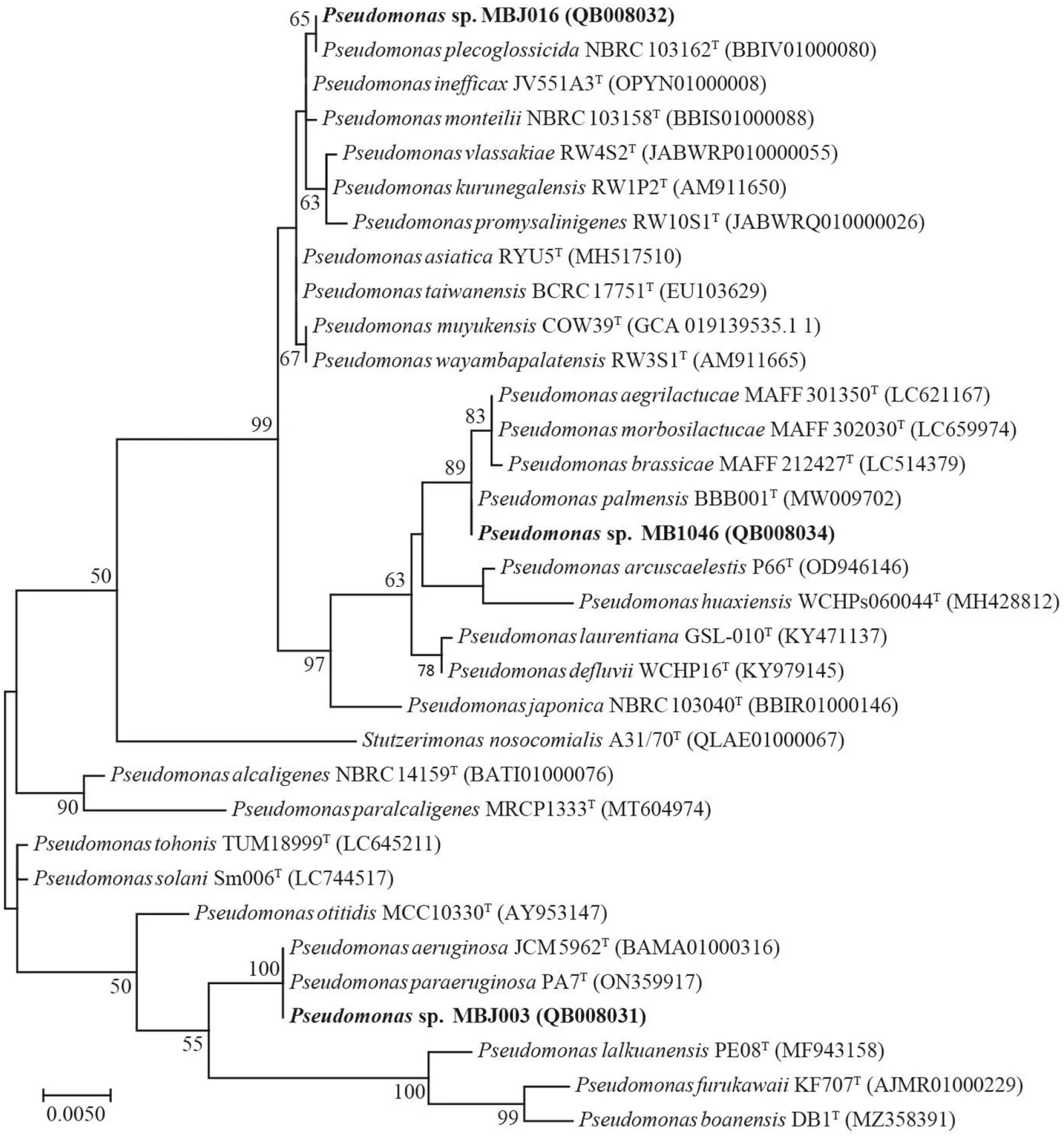
The ML phylogenetic tree of MBJ003, MBJ016, and MB1046 based on 16S rRNA gene sequences. Bar, 0.005 substitutions per nucleotide position. Bootstrap values > 50% are shown at branch points. Genbank accession numbers are given in parentheses.

### 3.2 Whole genome annotation and comparative genomic analysis of three *Pseudomonas* strains

The whole-genome sequences of MBJ003, MBJ016, and MB1046 were deposited in the NCBI database under the accession numbers JCCFZC000000000, JCCFZD000000000, and JCCFZE000000000, respectively. The draft genome sizes of MBJ003, MBJ016, and MB1046 were 6.4, 5.4, and 5.7 Mbp, with corresponding GC contents of 66.6%, 62.4%, and 64.3%. Detailed genomic characteristics were listed in Table 2.

**Table 2:** The genomic features of three *Pseudomonas* strains.

Genomic Features	MBJ003	MBJ016	MB1046
Size (Mbp)	6.4	5.4	5.7
No. of contigs	67	129	73
% GC content	66.6	62.4	64.3
No. of coding sequences	6,125	5,109	5,243
L50 (bp)	8	23	12
N50 (bp)	325,518	77,211	156,046
Number of RNAs	62	65	59
GenBank accession number	JCCFZC000000000	JCCFZD000000000	JCCFZE000000000

RAST annotation predicted 6125, 5109, and 5243 protein-coding genes, as well as 62, 65, and 59 RNA genes in the genomes of MBJ003, MBJ016, and MB1046, respectively. Hypothetical proteins accounted for 29.0%, 27.2%, and 28.1% of the total predicted proteins in the three strains. Functional subsystem analysis showed that the annotated proteins were mainly enriched in amino acid metabolism (470, 451, 468), carbohydrate metabolism (295, 229, 214), protein metabolism (220, 213, 211), cofactor and vitamin synthesis (197, 188, 180), membrane transport (143, 109, 79), fatty acid and lipid metabolism (132, 87, 96), respiration (117, 123, 109), nucleotide metabolism (103, 100, 98), and stress response (104, 104, 97) (Figure 3).

**Figure 3:**
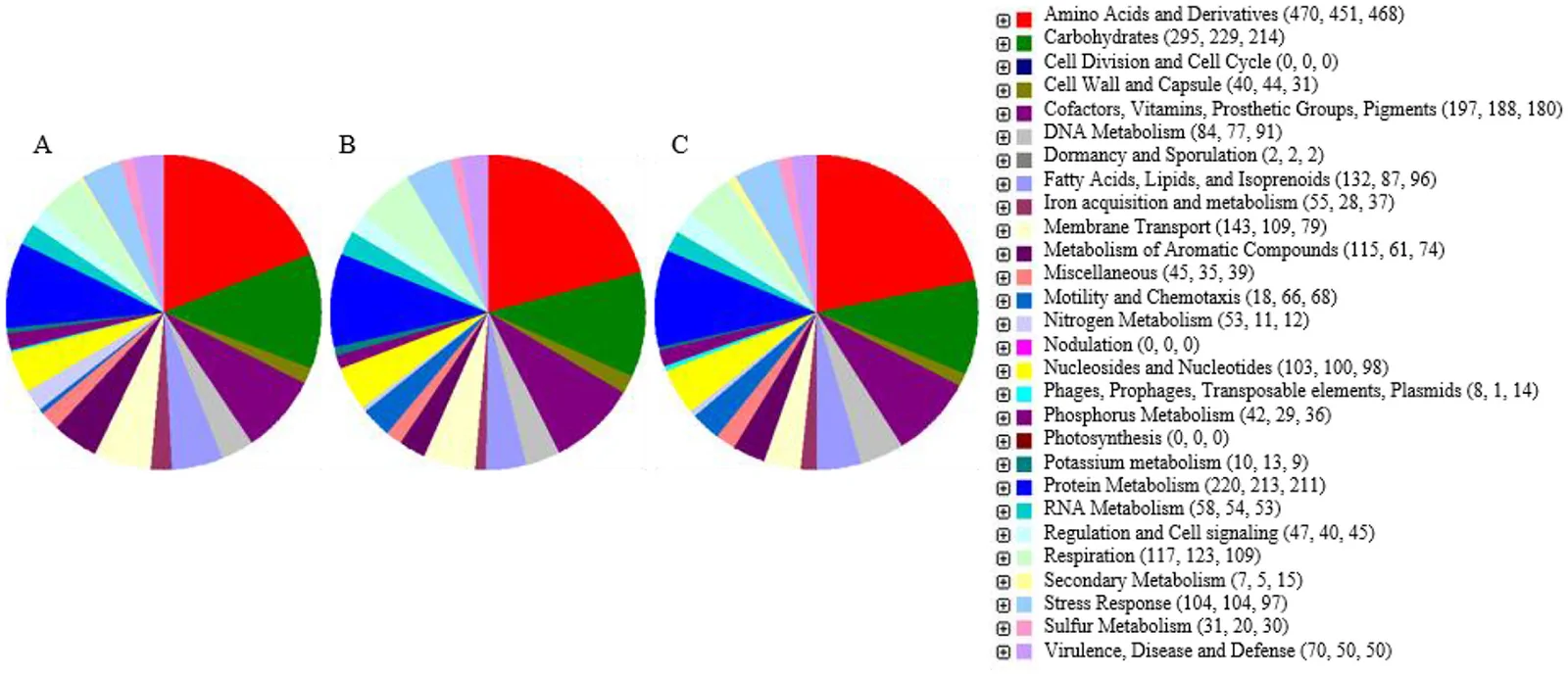
The genomic annotations of three *Pseudomonas* strains by RAST. **(A)** MBJ003, **(B)** MBJ016, and **(C)** MB1046.

The antiSMASH database prediction revealed a total of 15, 8, and 9 secondary metabolite BGCs in MBJ003, MBJ016, and MB1046, respectively. Four core BGCs (ripp-like3, NAGGN4, redox-cofactor, and Pf-5 pyoverdine) were conserved across all three strains. The pyoverdine SMX-1 was shared exclusively by MBJ003 and MB1046. Four BGCs, including pseudopaline, vibrioferrin, pseudomonine, and hydrogen cyanide, exhibited 100% sequence similarity with known functional BGCs. Seven BGCs showed extremely low homology ( ≤ 18%) with documented clusters, including streptophenazine 1/2, kutzneride 2, toxoflavin, MA026, lankacidin C, pf-5 pyoverdine, and deoxyhangtaimycin. In addition, nine BGCs were classified as novel, uncharacterized gene clusters (Table 3). Additionally, KEGG annotation revealed that strain MB1046 harbors an Imp/Vas secretion system (T6SS), which consists of 12 core components and 5 related proteins (Supplementary Table S3).

**Table 3:** The secondary metabolite gene clusters of three *Pseudomonas* strains predicted by antiSMASH databases.

Type	Most similar known cluster	BGC class	Similarity (%)	MBJ003	MBJ016	MB1046
Hserlactone1	Unknown		–	+	–	–
Phenazine	Streptophenazine 1/streptophenazine 2	Polyketide	6	+	–	–
Betalactone2, NRPS-like	Kutzneride 2	NRP	13	+	–	–
Opine-like-metallophore	Pseudopaline	Other	100	+	–	–
Phenazine	Toxoflavin	Other	18	+	–	–
Ripp-like3	Unknown		–	+	+	+
NRPS-like	MA026	NRP	5	+	–	–
Hserlactone	Unknown		–	+	–	–
NAGGN4	Unknown		–	+	+	+
Ripp-like	Unknown		–	+	–	–
Redox-cofactor	Lankacidin C	NRP + Polyketide	13	+	+	+
Hydrogen-cyanide, Nl-siderophore	Vibrioferrin	Other	100	+	–	–
NRPS	Pf-5 pyoverdine	NRP	7, 14	+	+	+
NRP-metallophore, NRPS	Pyoverdine SMX-1	NRP	41	+	–	+
NRP-metallophore, NRPS	Pyochelin	NRP	85	+	–	–
Acyl_amino_acids, RiPP-like	Unknown		–	–	+	–
RRE-containing5	Unknown		–	–	+	–
Nl-siderophore	Deoxyhangtaimycin	Polyketide + NRP	2	–	+	–
NRP-metallophore, NRPS, NRPS-like	Pseudomonine	NRPS	100	–	+	–
TransAT-PKS	Toblerol A-H	Polyketide	45	–	–	+
Hydrogen-cyanide	Hydrogen cyanide	Other	100	–	–	+
Betalactone	Unknown		–	–	–	+
LAP7	Unknown		–	–	–	+

+ indicated present; – indicated absent.

### 3.2 The three *Pseudomonas* strains exhibited plant growth-promoting traits

All three *Pseudomonas* strains exhibited plant growth-promoting traits, including inorganic phosphate solubilization, siderophore production, and IAA secretion (Supplementary Figure S2). After 3 d of incubation in liquid PKO media, the phosphate-solubilizing contents produced by strains MBJ003, MBJ016, and MB1046 were 48.4 ± 0.66 mg L^−1^, 50.6 ± 0.03 mg L^−1^ and 46.6 ± 0.11 mg L^−1^, respectively. When cultured in NB medium supplemented with 100 μg/mL L-tryptophan for 3 d, the IAA yields of MBJ003, MBJ016, and MB1046 reached 1.59 ± 0.22 μg mL^−1^, 6.43 ± 0.33 μg mL^−1^, and 10.63 ± 1.78 μg mL^−1^, respectively. Furthermore, strain MBJ003 exhibited prominent protease activity, with a value of 25.43 ± 1.23 U/mL after 3 days of cultivation (Table 4).

**Table 4:** The qualitative and quantitative determinations of plant-growth-promotion features of three *Pseudomonas* strains.

Test\strain	MBJ003	MBJ016	MB1046
Phosphorus solubilization (mg L^−1^)	48.4 ± 0.66	50.6 ± 0.03	46.6 ± 0.11
Potassium solubilization	–	–	–
Siderophore synthesis	+	+	+
IAA production (μg mL^−1^)	1.59 ± 0.22	6.43 ± 0.33	10.63 ± 1.78
Protease (U mL^−1^)	25.43 ± 1.23	–	–

+ indicates positive; – indicates negative.

### 3.3 The three *Pseudomonas* strains showed antagonistic activity against *G. citri-aurantii*

Plate confrontation assays revealed that strains MBJ003, MBJ016, and MB1046 exhibited significant inhibitory effects against *G. citri-aurantii* TSF19, with inhibition rates of 67.0%, 73.6%, and 64.4%, respectively (Figures 4A–D, I). In addition, the cell-free fermentation filtrates of the three *Pseudomonas* strains also suppressed growth of TSF19, with inhibition rates of 59.7%, 20.5%, and 16.1%, respectively (Figures 4E–H, J). These findings indicated that extracellular diffusible compounds of the three strains contribute to the antifungal activity.

**Figure 4:**
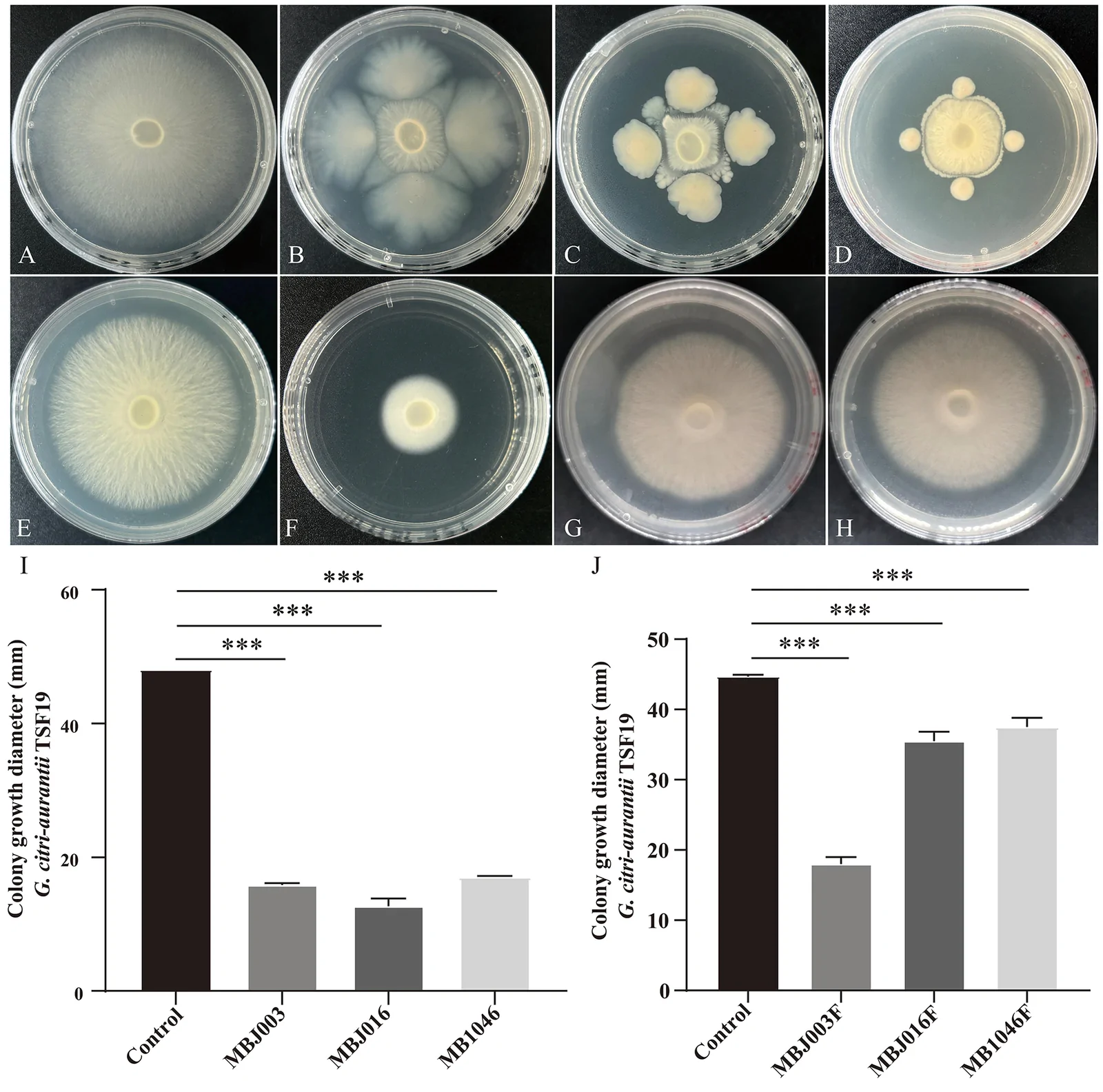
Antifungal activities of three *Pseudomonas* strains against *G. citri-aurantii* TSF19. **(A,E)** Growth of TSF19 on NA medium at 30 °C for 7 d **(A)** and on NA medium supplemented with 20% NB medium at 30 °C for 5 d **(E)**. **(B–D)** Antagonistic activities of MBJ003 **(B)**, MBJ016 **(C)**, and MB1046 **(D)** against TSF19 in plate confrontation assays. **(F–H)** Antagonistic effects of cell-free fermentation supernatants of MBJ003 **(F)**, MBJ016 **(G)**, and MB1046 **(H)** against TSF19; fungal colonies were cultured on NA medium containing 20% bacterial fermentation supernatant at 30 °C for 5 d. **(I,J)** Colony growth diameter of G. citri-aurantii TSF19 after plate confrontation with three Pseudomonas strains **(I)** and their fermentation supernatants **(J)**. Control, colony diameter of TSF19 without Pseudomonas treatment. *** indicates significant difference at the level of *p* < 0.001.

### 3.4 The three *Pseudomonas* strains exhibited high biocontrol efficacy against *G. citri-aurantii*

The detached citrus fruit assay was performed to assess the biocontrol efficacy of the three strains against citrus sour rot caused by *G. citri-aurantii*. Lesion area statistics showed that all three strains exhibited significant biocontrol effects on citrus sour rot (Figures 5A–H). Specifically, MBJ003 achieved the highest biocontrol efficacy of 76.9%, followed by MB1046 (68.0%) and MBJ016 (55.5%) (Figure 5I). These results indicated that the three strains have great potential as effective biocontrol agents for the management of citrus sour rot disease.

**Figure 5:**
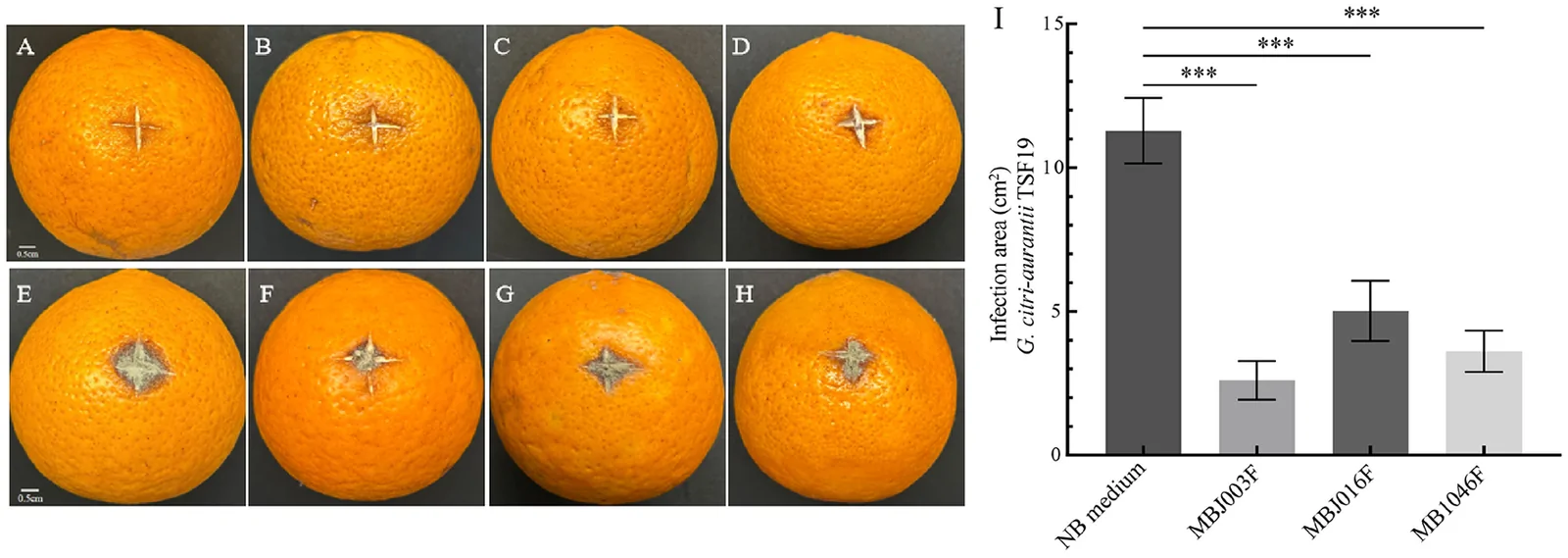
Biocontrol efficacy of three *Pseudomonas* strains against *G. citri-aurantii*. **(A–D)** Citrus fruits were immersed in sterile NB medium (control, **A**) or fermentation supernatants of MBJ003 **(B)**, MBJ016 **(C)**, and MB1046 **(D)**. **(E–H)** Lesion phenotypes of citrus fruits after inoculation with NB medium **(E)**, or fermentation supernatants of MBJ003 **(F)**, MBJ016 **(G)**, and MB1046 **(H)**. **(I)** Infection area of *G. citri-aurantii* TSF19 after inoculation with fermentation supernatants of three Pseudomonas strains, compared with NB medium control. *** indicates a significant difference at the level of *p* < 0.001.

### 3.6 The role of T6SS in strain MB1046 against *G. citri-aurantii*

To explore the role of the T6SS in the antifungal activity of strain MB1046, a deletion mutant Δ*vgrG* (IPAKHDLA_02735) with defective T6SS function was successfully constructed via homologous recombination (Figure 6A). The correctness of the double-crossover mutant was verified by gel electrophoresis (Figure 6B). Subsequently, co-cultivation assays were performed by incubating WT_MB1046_ or Δ*vgrG* cells with TSF19 conidia on NA plates to simulate direct bacterial–fungal cell contact. The results showed that the antifungal ability of the Δ*vgrG* was markedly impaired, and it almost failed to inhibit the growth of TSF19 compared with WT_MB1046_ (Figures 6C, D). A parallel spotting assay with a 1.0 cm spacing between bacterial and fungal spots further confirmed the phenotypic difference. The Δ*vgrG* exhibited significantly weaker inhibitory activity against TSF19 than WT_MB1046_ (Figures 6E, F). Furthermore, a physical separation assay using a 0.22 μm PVDF filter membrane was conducted to block direct cell contact. When bacterial cells and fungal conidia were completely separated by the filter, the Δ*vgrG* lost its antifungal activity. In contrast, WT_MB1046_ still retained partial inhibitory effects against fungal growth even without direct cell contact (Figures 6G, H). Taken together, we speculate that strain MB1046 may exert antifungal activity via the T6SS in a cell-contact-dependent manner.

**Figure 6:**
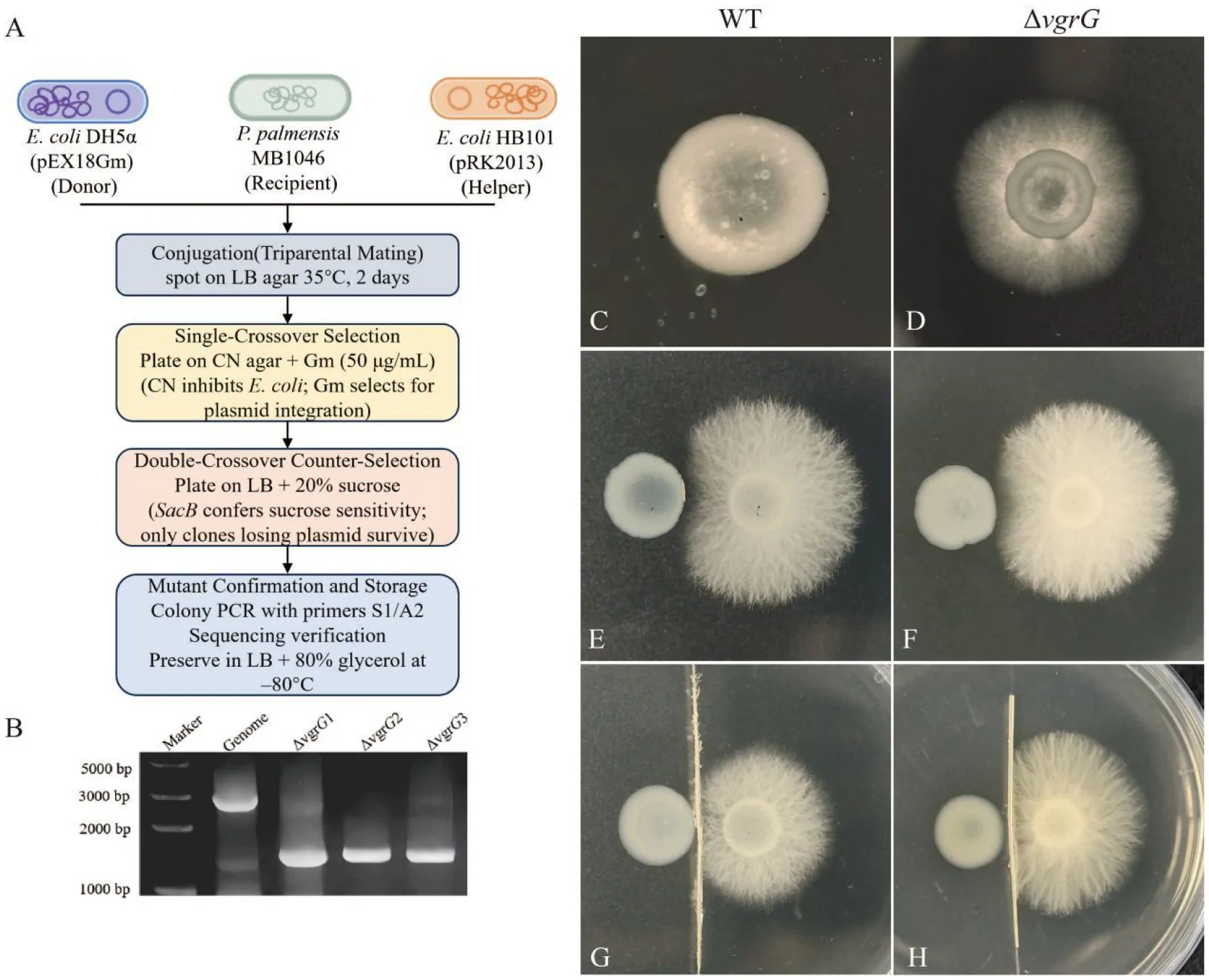
Construction and functional verification of the Δ*vgrG*. **(A)** Schematic diagram of the Δ*vgrG* construction strategy. **(B)** Gel electrophoresis identification of the double-crossover Δ*vgrG*. **(C,D)** Antifungal phenotypes of WT_MB1046_ **(C)** and T6SS-deficient mutant (Δ*vgrG*) **(D)** against TSF19. **(E,F)** Confrontation assays of WT_MB1046_ **(E)** and Δ*vgrG* **(F)** against TSF19. **(G,H)** Contact-dependent antifungal verification assays. WT_MB1046_ **(G)**, Δ*vgrG* **(H)** and TSF19 were separated from TSF19 by 0.22 μm PVDF filter membrane.

## 4 Discussion

In the present study, three *Pseudomonas* strains, *P. paraeruginosa* MBJ003, *Pseudomonas* sp. MBJ016 and *P. palmensis* MB1046, were systematically verified to possess multiple plant growth-promoting properties, including phosphate solubilization, siderophore synthesis, and IAA production. These core metabolic activities enable rhizosphere bacteria to effectively improve soil nutrient availability, regulate plant endogenous hormone homeostasis, and ultimately promote the growth and biomass accumulation of host plants (Goswami et al., 2016; Waadt et al., 2022). In addition to multiple growth-promoting traits, the three *Pseudomonas* strains displayed prominent biocontrol performance, effectively inhibiting the pathogen *G. citri-aurantii* and exerting significant suppressive effects on citrus sour rot. These findings confirm that the three *Pseudomonas* strains are multifunctional PGPR resources integrating growth promotion and disease resistance, with great application potential in vegetable and citrus green cultivation.

Notably, antimicrobial activity was detected in the fermentation supernatants of all three *Pseudomonas* strains, indicating that extracellular soluble metabolites are active substances involved in pathogen inhibition. Combined with whole-genome sequencing analysis, a large number of functional gene clusters responsible for secondary metabolite biosynthesis were annotated in their genomes, including ripp-like3, NAGGN4, redox-cofactor, Pf-5 pyoverdine, pseudopaline, vibrioferrin, pseudomonine, and hydrogen cyanide (HCN). Additionally, multiple antimicrobial secondary metabolite clusters, such as streptophenazine 1/2, kutzneride 2, toxoflavin, MA026, lankacidin C, and deoxyhangtaimycin, were also founded. Most of these compounds have been proven to exert broad-spectrum antimicrobial and antifungal activities via distinct modes of action: siderophore derivatives including pyoverdine, vibrioferrin, and pseudomonine inhibit pathogen growth by competing for rhizosphere iron nutrition (Ghssein and Ezzeddine, 2022; Matthijs et al., 2009; Stanborough et al., 2018); HCN disrupts the respiratory electron transport chain of phytopathogens and induces oxidative stress (Karthika et al., 2020); phenazine and toxoflavin compounds damage pathogen cell membrane integrity and interfere with energy metabolism (Adrien and Martin, 2018); while lankacidin and other cyclic peptide metabolites suppress pathogen proliferation by inhibiting protein synthesis and cell wall formation (Auerbach et al., 2010). The presence of multiple secondary metabolite biosynthesis gene clusters indicates that the three *Pseudomonas* strains possess the potential to exert antifungal activity by producing antimicrobial secondary metabolites.

In addition to antimicrobial secondary metabolites, some bacteria protect plants from filamentous fungal infections through T6SS-mediated intercellular interactions (Lin et al., 2025). We further revealed the role of the T6SS in the inhibition of *G. citri-aurantii* by strain MB1046. Our findings are consistent with previous observations that *Pseudomonas fluorescens* 2P24 and *Pseudomonas fulva* FoE9 utilized the T6SS as an antifungal device (Lin et al., 2025). Because the complemented Δ*vgrG* strain was not generated in the present study, the result remains to be further verified. Prior study also indicated that some bacterial T6SS effector proteins showed *in vitro* antifungal activity to filamentous fungi (Nachmias et al., 2025). Additionally, T6SS and its effectors were also reported to act against gram-negative bacteria (Le et al., 2021).

Collectively, this study clarifies plant growth-promoting traits and biocontrol mechanism of three *Pseudomonas* strains against *G. citri-aurantii*. Extracellular fermentation metabolites and T6SS-mediated cell-contact-dependent antifungal effects may constitute the synergistic antifungal strategy of *Pseudomonas* strains. Future research can further isolate the antifungal metabolites, verify the expression levels and functional activities of key secondary metabolites during rhizosphere colonization, explore the synergistic antibacterial effects among different metabolites, and optimize the fermentation and application conditions of the strains. These efforts will lay a theoretical foundation for the development of efficient and stable microbial inoculants for citrus and vegetable disease prevention and growth promotion, and support the sustainable development of ecological agriculture.

## Data Availability

The datasets presented in this study can be found in online repositories. The names of the repositories and accession numbers can be found in the article and Supplementary materials.
