# Supplementary material for: Plant growth-promoting traits and biocontrol mechanism of three Pseudomonas strains against Geotrichum citri-aurantii
Source: Front Microbiol. 2026 Oct 26;17:1970166. doi: 10.3389/fmicb.2026.1970166 (PMC13745667; doi:10.3389/fmicb.2026.1970166)
Supplement: Supplementary file 1 [file Supplementary_file_1.docx]

**Supplementary data**

**Plant growth-promoting traits and biocontrol mechanism against Geotrichum citri-aurantii of three *Pseudomonas* strains**

Chunling Wang, Lutian Mao*, Boshi Zhu, Xing Liu, Xinjian Xu, Jiaxin Zhuo

College of Life Science, Huizhou University

**Corresponding author:** mlt@hzu.edu.cn

OD600

| Time (h) | MBJ003 | MBJ016 | MB1046 |
| --- | --- | --- | --- |
| 0 | 0.00533333 | 0.007 | 0.008 |
| 4 | 0.114667 | 0.261667 | 0.297 |
| 8 | 0.695667 | 0.877333 | 0.844 |
| 12 | 1.18267 | 1.41367 | 1.48067 |
| 24 | 1.84267 | 1.77733 | 1.89833 |
| 36 | 1.89267 | 1.96533 | 2.14167 |
| 48 | 1.94267 | 2.08233 | 1.92833 |

FIGURE S1. The growth curves of three *Pseudomonas* strains in NB media.

FIGURE S2. Plant growth-promoting activities of three *Pseudomonas* strains. (A-C) Dissolved inorganic phosphorus activities of MBJ003 (A), MBJ016 (B) and MB1046 (C); (D-F) Siderophore qualitative detection of MBJ003 (D), MBJ016 (E) and MB1046 (F) on the CAS media.

Table S1. The strains, plasmids and primers used in this study.

| **Strains** | **Characteristics** | **Source** |
| --- | --- | --- |
| MB1046 | *Pseudomonas palmensis* MB1046 wildtype | Isolated from tomato rhizosphere soil |
| *∆vgrG* | In-frame deletion of vgrG in MB1046 | This study |
| pEX18Gm | Suicide vector with a sacB gene, GmR | Lab collection |
| pRK2013 | Helper plasmid was used to provide the genes encoding the conjugation machinery | Lab collection |
| vgrG-S1 | CAGCTATGACATGATTACGAATTCGCCTTCACCGCCGATTCGGTCG | This study |
| vgrG-R1 | CCGATGTTCCTCCCGCTGCAAA | This study |
| vgrG-S2 | TTTGCAGCGGGAGGAACATCGGCCCTGGCCACGGTCAAGAGCAA | This study |
| vgrG-R2 | CCTGCAGGTCGACTCTAGAGGATCCGCGGCTCGGGGAAGGTCCCGGC | This study |

Table S2. The ANI and dDDH values of strain MB1046 and its related species of the genus *Pseudomonas*.

| **Code** | **Most Related Species** | **ANI (%)** | **dDDH (%)** |
| --- | --- | --- | --- |
| 1 | *Pseudomonas plecoglossicida* NBRC 103162^T^ | 86.9 | 33.0 |
| 2 | *Pseudomonas inefficax* JV551A3^T^ | 93.2 | 52.6 |
| 3 | *Pseudomonas monteilii* NBRC 103158^T^ | 85.5 | 40.1 |
| 4 | *Pseudomonas asiatica* RYU5^T^ | 93.7 | 55.1 |
| 5 | *Pseudomonas taiwanensis* BCRC 17751^T^ | 85.5 | 30.9 |
| 6 | *Pseudomonas promysalinigenes* RW10S1^T^ | 85.0 | 29.7 |
| 7 | *Pseudomonas alloputida* Kh7^T^ | 89.3 | 38.6 |
| 8 | *Pseudomonas wayambapalatensis* RW3S1^T^ | 84.6 | 29.2 |
| 9 | *Pseudomonas kurunegalensis* RW1P2^T^ | 89.7 | 40.0 |
| 10 | *Pseudomonas vlassakiae* RW4S2^T^ | 86.7 | 33.4 |
| 11 | *Pseudomonas entomophila* L48^T^ | 85.4 | 30.5 |
| 12 | *Pseudomonas juntendi* BML3^T^ | 88.0 | 35.7 |
| 13 | *Pseudomonas fakonensis* COW40^T^ | 85.4 | 30.5 |
| 14 | *Pseudomonas xanthosomatis* COR54^T^ | 85.5 | 30.8 |
| 15 | *Pseudomonas xantholysinigenes* RW9S1A^T^ | 85.4 | 30.5 |
| 16 | *Pseudomonas mosselii* CIP 105259^T^ | 85.5 | 30.7 |
| 17 | *Pseudomonas peradeniyensis* BW13M1^T^ | 85.8 | 31.1 |
| 18 | *Pseudomonas anuradhapurensis* RD8MR3^T^ | 90.2 | 41.8 |
| 19 | *Pseudomonas kermanshahensis* SWRI100^T^ | 87.2 | 33.8 |
| 20 | *Pseudomonas oryzicola* RD9SR1^T^ | 90.3 | 42.1 |
| 21 | *Pseudomonas sichuanensis* WCHPs060039^T^ | 85.8 | 30.6 |
| 22 | *Pseudomonas urmiensis* SWRI10^T^ | 84.4 | 28.8 |
| 23 | *Pseudomonas shirazensis* SWRI56^T^ | 82.7 | 27.0 |
| 24 | *Pseudomonas soli* F-279_208^T^ | 85.1 | 30.1 |
| 25 | *Pseudomonas parafulva* NBRC 16636^T^ | 85.5 | 30.6 |
| 26 | *Pseudomonas guariconensis* LMG 27394^T^ | 84.3 | 28.7 |
| 27 | *Pseudomonas maumuensis* COW77^T^ | 85.3 | 30.2 |
| 28 | *Pseudomonas fortuita* GMI12077^T^ | 89.3 | 38.8 |
| 29 | *Pseudomonas putida* NBRC 14164^T^ | 89.8 | 40.0 |
| 30 | *Pseudomonas farsensis* SWRI107^T^ | 84.0 | 28.8 |
| 31 | *Pseudomonas japonica* NBRC 103040^T^ | 81.8 | 25.5 |
| 32 | *Pseudomonas defluvii* WCHP16^T^ | 79.9 | 24.0 |
| 33 | *Pseudomonas fulva* NBRC 16637^T^ | 83.4 | 27.9 |
| 34 | *Pseudomonas sessilinigenes* CMR12a^T^ | 79.0 | 23.2 |
| 35 | *Pseudomonas alkylphenolica* KL28^T^ | 81.3 | 25.0 |

Table S3. The T6SS components of strain MB1046.

| **Gene Locus Numbers** | **Type VI secretion system** |
| --- | --- |
|  | **Imp/Vas secretion system core components** |
| IPAKHDLA_03919 | K11891 impL, vasK, icmF; type VI secretion system protein ImpL |
| IPAKHDLA_03918 | K11892 impK, ompA, vasF, dotU; type VI secretion system protein ImpK |
| IPAKHDLA_03917 | K11893 impJ, vasE; type VI secretion system protein ImpJ |
| IPAKHDLA_03911 | K11895 impH, vasB; type VI secretion system protein ImpH |
| IPAKHDLA_03910 | K11896 impG, vasA; type VI secretion system protein ImpG |
| IPAKHDLA_03908 | K11900 impC; type VI secretion system protein ImpC |
| IPAKHDLA_03907 | K11901 impB; type VI secretion system protein ImpB |
| IPAKHDLA_02735 | K11904 vgrG; type VI secretion system secreted protein VgrG |
| IPAKHDLA_03909 | K11905 type VI secretion system protein |
| IPAKHDLA_03916 | K11906 vasD, lip; type VI secretion system protein VasD |
| IPAKHDLA_03912 | K11907 vasG, clpV; type VI secretion system protein VasG |
| IPAKHDLA_03906 | K11910 vasJ; type VI secretion system protein VasJ |
|  | **Imp/Vas secretion system related proteins** |
| IPAKHDLA_03915 | K11894 impI, vasC; type VI secretion system protein ImpI |
| IPAKHDLA_03913 | K11914 sfa2; sigma-54 dependent transcriptional regulator |
| IPAKHDLA_03920 | K11915 stp1, pppA; serine/threonine protein phosphatase Stp1 [EC: stp1, pppA; serine/threonine protein phosphatase Stp1…3.1.3.16] |
| IPAKHDLA_03921 | K11916 stk1; serine/threonine-protein kinase Stk1 [EC:2.7.11.-] stk1; serine/threonine-protein kinase Stk1 [EC:2.7.11] |
| IPAKHDLA_03306 | K11917 sfa3; sigma-54 dependent transcriptional regulator |
